# Ethical, legal, and social aspects of health technologies for home-based paediatric palliative care – a systematic review

**DOI:** 10.1186/s12904-025-01774-7

**Published:** 2025-05-16

**Authors:** Erik Bjørnerud, Simen A. Steindal, Bjørn M. Hofmann, Anette Winger, Kirsti Riiser, Weiqin Chen, Heidi Holmen

**Affiliations:** 1 Department of Natural Sciences, Practical-Aesthetic, Social and Religious Studies, Faculty of Teacher Education and Languages, Østfold University College, Fredrikstad, Norway; 2 Lovisenberg Diaconal University College, Oslo, Norway; 3 Institute of Nursing, Faculty of Health, VID Specialized University, Oslo, Norway; 4 Department of Health Sciences, Norwegian University of Science and Technology (NTNU), Trondheim, Norway; 5 Centre of Medical Ethics, University of Oslo, Oslo, Norway; 6 Department of Nursing and Health Promotion, Faculty of Health Sciences, Oslo Metropolitan University, Oslo, 0130 Norway; 7 Department of Rehabilitation Science and Health Technology, Faculty of Health Sciences, Oslo Metropolitan University, Oslo, 0130 Norway; 8 Department of Computer Science, Faculty of Design, Oslo Metropolitan University, Oslo, 0130 Norway; 9 Intervention Centre, Oslo University Hospital, Oslo, 4950 Norway

**Keywords:** E-health, Ethical, legal, and social aspects, Home-based health technologies, Paediatric palliative care, Systematic review, Telehealth

## Abstract

**Background:**

Home-based health technologies for paediatric palliative care have great potential to improve care for children, caregivers, healthcare professionals, and health systems. However, no systematic reviews have directly addressed the intersections among the ethical, legal, and social aspects of these technologies for paediatric palliative care. The objective of this systematic review was to identify and analyse the ethical, legal, and social aspects of health technologies for home-based paediatric palliative care.

**Methods:**

We have conducted a systematic review, inspired by the framework suggested by McCullough. We registered the review protocol in PROSPERO (CRD42024496034) and conducted a systematic search in six databases (ASSIA, Cinahl, Embase, Medline, PsycInfo, and Web of Science) on 27 November 2023 to identify relevant studies. Pairs of authors independently assessed the eligibility of the studies and extracted data. The eligible studies employed a range of different methods from randomised controlled trials to usability studies. We then synthesised the data according to the ethical, legal and social aspects of the technologies.

**Results:**

Overall, our search resulted in 9,545 reports, which were screened after deduplication. The quality of the reports was assessed according to being published in peer reviewed journals. Fifteen reports were included, which showed that the main ethical issues are harm reduction, improved services, agency and autonomy, trust and empowerment. The main legal aspects are privacy equal access to care, participation in decisions and standardisation. The main social issues are cost reduction, transformation of family relations and novel modes of communication. Health technologies have the potential to alleviate burdens and improve the quality of care for children in paediatric palliative care and their families, but they also create novel burdens through constant reporting requirements and the vulnerability of some health technologies to technological malfunction. Nevertheless, they can increase family inclusion and children’s autonomy and participation, thus empowering children, particularly through co-development of solutions. Furthermore, studies have indicated that health technologies themselves may have positive effects on children’s health. The legal aspects of health technologies pertain to privacy and control over one’s health information and equitable access to care and participation in care, while social issues can potentially reduce costs for health systems but also involve novel costs.

**Conclusion:**

The reviewed studies concerning the co-development of health technologies reported increased benefits in terms of health, agency, well-being, and strengthened children’s rights in home-based paediatric palliative care. However, the social dimensions of such technologies can lead to both public savings and reconfiguration of family constellations. We recommend that future researchers consider privacy, the formal dimensions of apps and smartphones, and their impacts on families.

**PROSPERO reference:**

CRD42024496034.

**Supplementary Information:**

The online version contains supplementary material available at 10.1186/s12904-025-01774-7.

## Background

PPC aims to improve or preserve the quality of life (QoL) of children with life-limiting (LL) or life-threatening (LT) conditions and their families [1]. All children with LL or LT conditions should be offered PPC [2], which is oriented towards children’s often complex needs, including their clinical, psychological, social, spiritual, family, communication, and ethical needs [2].

A central tenet of PPC is that children should be cared for at home whenever possible [2] and home is often the preferred place for families to receive care [3]. However, home-based PPC creates many challenges for children, their families, and healthcare services, including struggles in coordinating care for children and securing appropriate support from adequately skilled staff with relevant experience [1]. Home-based health technologies, alone or in combination, can address and alleviate such challenges [4, 5]. Despite various understandings of what constitutes telehealth, medical technologies, health technologies, and assistive technologies [6], ‘health technologies’ is a term describing a high-order phenomenon encompassing and including all digital technologies introduced and applied to care for children in PPC [7]. Bradford et al. [4], in a systematic review that had a primary orientation towards PPC but also included adult focussed studies, found that different forms of health technology solutions had no negative effects and could have positive effects on QoL and anxiety. In addition, health technologies may increase access to care if families reside far from healthcare services, and they can also increase collaboration between families and HCPs. Furthermore, they allow children to be more active participants in their own care and decisions regarding themselves. They can also facilitate the visualisation of health characteristics or needs and the details of treatments or procedures, thus enhancing the quality of care [4, 8, 9]. However, home-based health technologies can be burdensome if they malfunction, do not meet the needs of children and their families, and/or pose challenges in meeting privacy regulations during their use for home-based PPC [4, 8, 9]. Moreover, health technologies may have unforeseen consequences—positive or negative [10].

Although attention has been paid to people’s experiences and the outcomes of these technologies, there have been calls for greater consideration of their ethical, legal, and social aspects (ELSA). These aspects have been central to the overall field of health technologies for more than two decades [11–14]. ELSA emerged in the 1990s and was initially connected to emerging science and technology fields, such as genetic modification, nanotechnology, brain research, and precision medicine [15–18]. However, more recently, there has been a move towards more specific applications [19, 20]. The core aim of ELSA studies is to integrate the three previously distinct ethical, social, and legal disciplines that were studied in silos and to perceive the interplays among them [18]. These studies have been so successful that we now experience difficulties separating these previously clearly differentiated spheres [21]. The social dimension of PPC typically relates to welfare and economic factors, the legal dimension relates to issues of justice and fairness, and the ethical dimension comprises four principles—the best interest, risk–benefit proportionality, distributive justice, and autonomy principles [2]. There are concerns cutting across the ethical, social, and legal disciplines, such as privacy, information security, and how to integrate the above-mentioned changes into healthcare systems and the evaluation of such interventions [22, 23]. Thus, an ELSA framework appears relevant for addressing the normative issues involved in PPC.

**Fig. 1:**
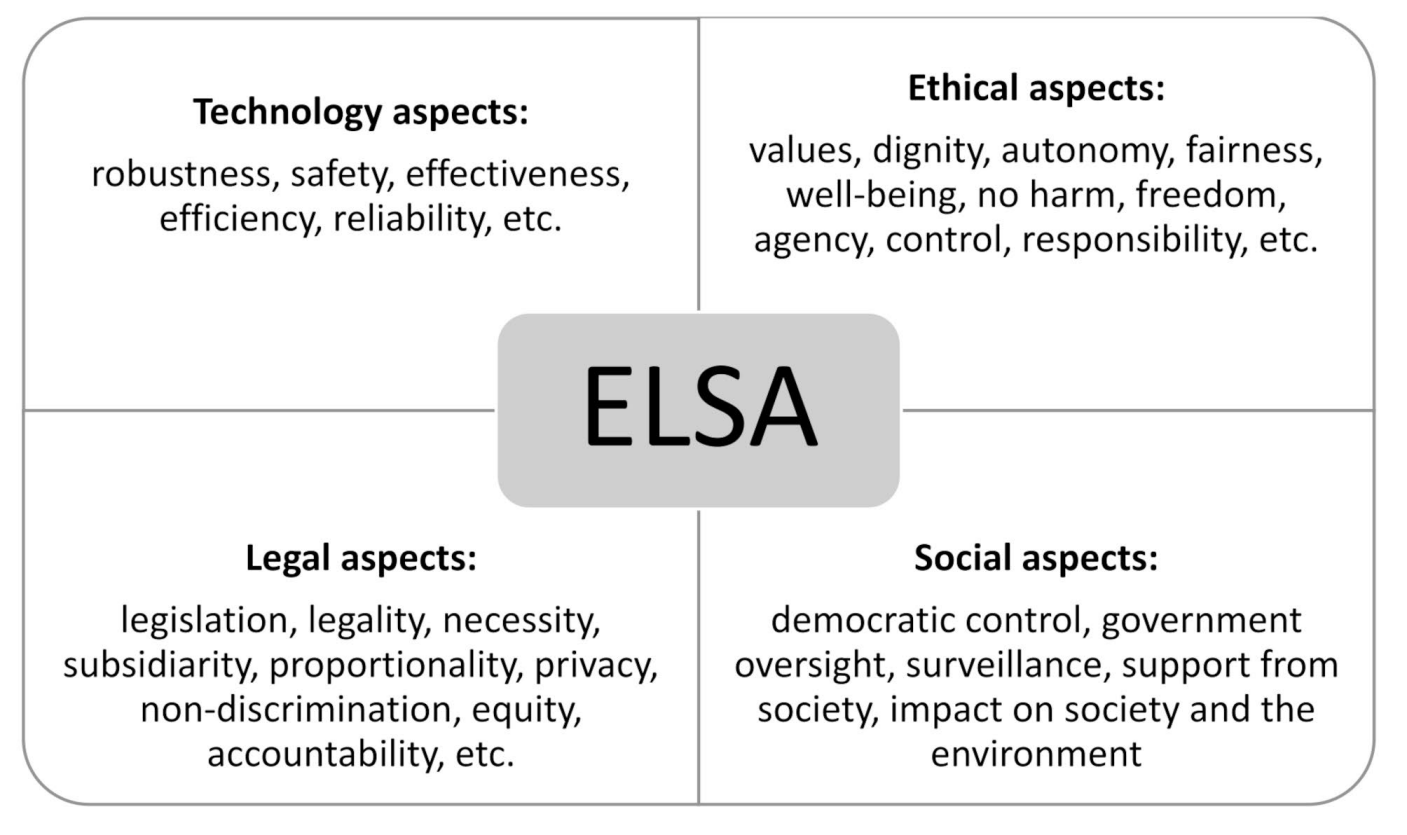
‘ELSA in a box’ [24]

Many reviews have considered ELSA in home-based health technologies, especially those designed for older adults who wish to continue living at home [25, 26]. These studies have typically focused on privacy, autonomy, equal access, anti-discrimination (ageism), trust, stigma, the medicalisation of the home, human vs. machine interactions, individual users vs. the general image of a user, and responsibility [27–29].

There has not been any reviews on ELSA in PPC, but some reviews have addressed aspects of ELSA in adult palliative care [30–33]. Demiris et al. [30, 31] examined issues including information security, informed consent, equal access, autonomy vs. dependence, the lack of human touch, the medicalisation of the home, and the usability of telehealth solutions in adult palliative care. In addition, the moral relevance of the family and the challenges of articulating this relevance have received attention [34, 35]. Recently, Steindal et al. [33] discussed the importance of making autonomous choices, achieving psychological and physiological relief, collecting and presenting meaningful data to users, and equity in adapting health technology solutions to users.

Currently, there is significant concern regarding the ELSA of children’s interactions with digital online technologies [36, 37], and some attention has been paid to the use of technologies for gamifying healthy behaviours among children [38] or facilitating participation and communication [39]. In a discussion over ethical principles for digital PPC, Garani-Papadatos et al. [32] considered rights such as autonomy, privacy, fairness, and well-being.In addition, some studies on paediatric home-based health have addressed issues of access, quality, and family-centricity, but they have paid less attention to effectiveness, efficiency, safety, and equity [40].

In the field of palliative care, much attention has been oriented towards HCPs. In a systematic review of HCPs’ practical experiences of ethical challenges in specialist palliative care, Schofield et al. [41] identified challenges in applying the principles of autonomy, dignity, and equity. Furthermore, the researchers found that in delivering care to patients and their families, HCPs experienced value conflicts with both their institutions and the wider society. Regarding health technologies for home-based PPC, HCPs have claimed that the value of care and services is influenced by technology [7]. Furthermore, the development and testing of health technologies poses a dilemma in ensuring the best possible care for all children while conducting randomised controlled trials (RCTs) [8]. However, no systematic reviews have directly addressed the intersections among ELSA of health technologies for home-based PPC. Children have a right to participate in society—and, consequently, in research [42]—but with due attention to the ethics and provision of proper care for children [32].

## Objective

The objective of this systematic review was to analyse the ELSA of health technologies for home-based PPC based on the following research questions:

1. What are the ELSA of health technologies for home-based PPC?
2. What are their implications for the future development of digital solutions for home-based PPC?

## Methods

In this systematic review, we took as a point of departure the framework developed by McCullough et al. [43]: We modified point 3) to “Assess the adequacy of the ethical issues of the reports identified”, which will be the topic of our discussion. The four stages are thus:

1. Identify a focused question.
2. Conduct a literature search using key terms relevant to the focused question.
3. Assess the adequacy of the ethical issues of the reports identified.
4. Identify the conclusions drawn in each report and whether they apply to the focused question.

Before performing the review, we searched for similar reviews in the International prospective register of systematic reviews (PROSPERO) and registered the review protocol in PROSPERO (CRD42024496034). Deviations from the protocol are described in Supplementary File 2. The review is reported according to the Preferred Reporting Items for Systematic reviews and Meta-Analyses (PRISMA [44]).

### Eligibility criteria

Table 1 shows the eligibility criteria based on the population, concept, and context framework [45] as well as the type of study, language, and study period.

**Table 1:** Description of eligibility criteria

Criterion	Inclusion	Exclusion
Population	Children aged 0–18 years with a LL and/or LT conditions in need of PPC, their families, HCPs, social workers, and teachers.	Adults aged 19 years or older, children with chronic or long-term illnesses not in need of PPC, and cancer survivors.
Concept	Use of health technologies for PPC with or without interactions between children and/or families with healthcare or social care providers or teachers. Relevant papers need to include ELSA (i.e. papers pertaining to explicit value discussions, official legal frameworks, and/or ties to social institutions outside the home) that are reported as findings.	
Context	Home-based care. A home may also be an institution that is regarded as the child’s home.	Outside home-based care.
Types of literature	Empirical studies regardless of design published in peer-reviewed journals.	Any reviews, conference abstracts, conference proceedings, study protocols, guidelines, position papers, discussion/theoretical papers, reports, PhD or master’s theses, letters, comments, editorials, books, or book chapters.
Language	Studies limited to papers published in the Danish, English, Norwegian, Swedish, French, and Italian languages.	
Time period	Studies published between January 2013 and November 2023	

The choice of languages was based on the authors’ understanding of these languages. We chose this period to identify up-to-date, relevant ELSA of health technologies that can inform future research and the development of health technologies and services.

### Search strategy

We performed a comprehensive search on 27 November 2023 in the ASSIA, Cinahl, Embase, Medline, PsycInfo, and Web of Science databases pertaining to health, children, and health technologies, which were the focus of this review. The search strategy was developed in Medline by two academic librarians (Elisabeth Karlsen & Ingjerd L. Ødemark) with expertise in systematic searches of medical research databases in collaboration with the research team members EB, SAS, and HH. EB and SAS piloted the search strategy, and the final search is described in Supplementary File 3.

We excluded grey literature because our aim was to identify and explore only peer-reviewed published studies.

### Selection of studies

We transferred the identified publications to EndNote to remove duplicates and then transferred them to the Covidence webtool [46]. Covidence ensured blinding of the study selection process and that two authors independently assessed all the publications. Seven authors screened the titles and abstracts independently, and conflicts among the authors were resolved by EB and HH. In the second assessment round, the same authors independently read the full texts of the publications. Again, conflicts among the authors were resolved by the same authors.

### Data extraction

There are no established methods for ELSA studies or ethics for related data extraction [47]. As a point of departure, we took the questions developed by Hofmann et al. [48] for the assessment of health technologies according to their purposes understood as communication, compensation, help in everyday tasks, monitoring, treatment, entertainment and social support. To ensure coherence in the data extraction process, we took inspiration from a recently developed overview of the field, as illustrated in Fig. 1 [24].

The authors HH and EB collected all the reports for the full-text review and read them. First, EB established a data extraction table with specific report identifiers, including the study number, author(s), year, country, type of study, participants, methodology used, and the ELSA identified and discussed in the reports. The included studies were primary ELSA studies regarding health technologies for home-based PPC (not general studies on health technologies for home-based PPC). EB extracted data from the results, discussion, and/or conclusion sections of the reports, while HH checked the data accuracy against the reports.

### Quality appraisal

Quality appraisals of ELSA studies are challenging due to the diversity of accepted methods and disciplinary paradigms. We applied the so-called satisficing approach which has as its quality indicator that the studies are published in channels with an editor(ial team) and academic peer review [47, 49]. All of the studies in this review met this criterion.

### Synthesis methodology

The synthesis for this review was informed by the ELSA framework. In line with other studies, we did not establish specific a priori themes prior to the data extraction [19, 50, 51]. Rather, EB and HH read the full papers, using the ELSA in a box framework (Fig. 1) [24] as a heuristic, and we identified ELSA-relevant assumptions, practices, findings, and conclusions and placed them under separate headings for ‘Ethical’, ‘Social’, and ‘Legal’ issues. Consequently, the ELSA perspective provided the framework for a deductive analysis. In the next step, we identified commonalities among the findings under these three headings. Based on the data in each aspect we inductively redefined and revised the name of the themes. Thus, our analysis leaned towards an inductive practice, as we considered both unique and generalisable findings in each report. We presented a preliminary analysis at a meeting with all the authors present, at which we discussed the process and findings. When the analysis had been completed, each of the three ELSA categories was described in a synthesised text for presentation, overseen by BH. To the fullest extent possible, we assigned these findings to well-known topics (avoid or reduce harm, benefits, agency, autonomy, empowerment, privacy, just or fair care, and beneficence) based on a discussion of the respective disciplines [52] as presented in the introduction to this article.

## Results

### Publication selection process

#### Characteristics of the included reports

**Fig. 2:**
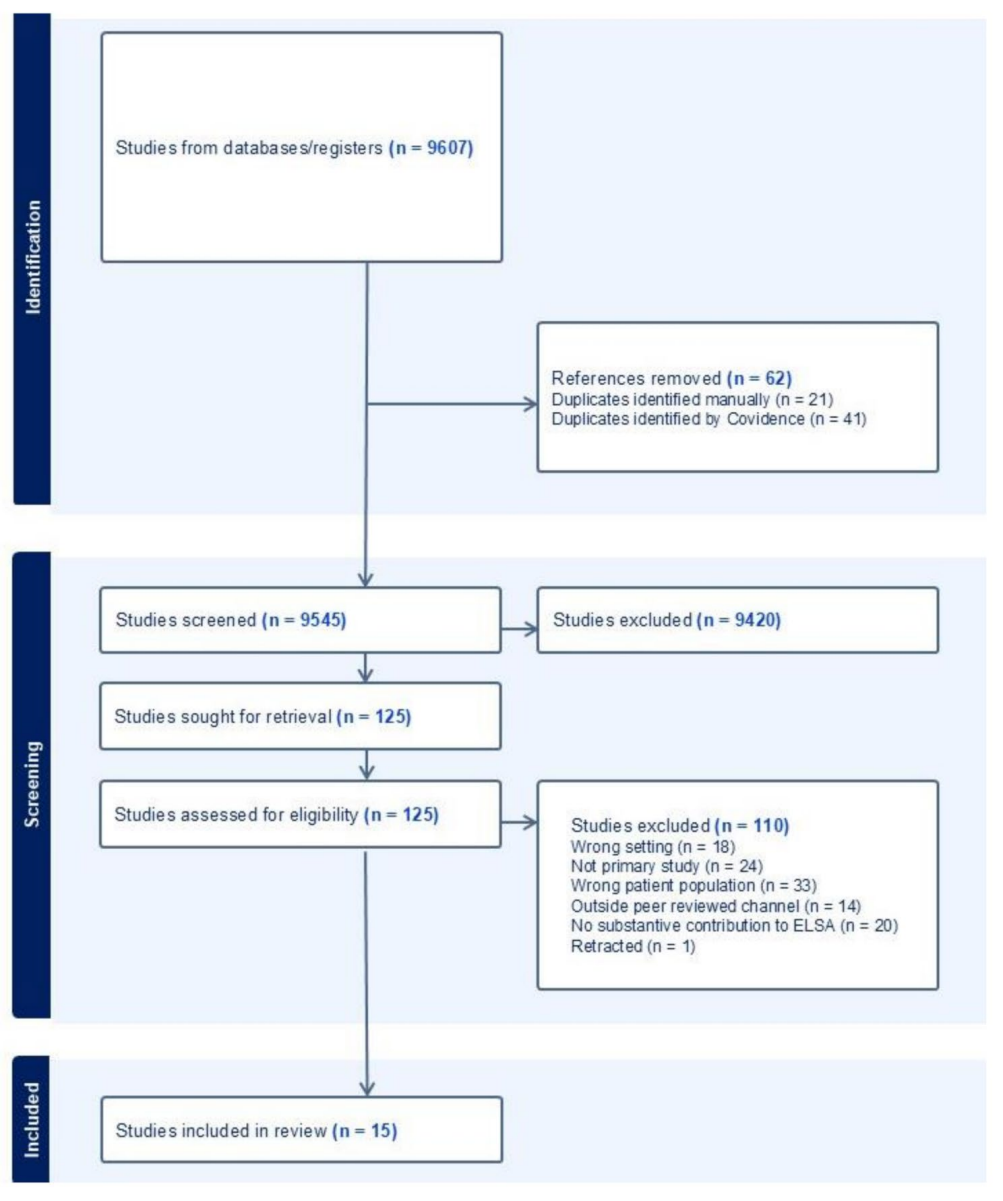
Flowchart of the search and screening process

We identified 15,616 citations through searches (Fig. 2). After deduplication, 9,545 reports remained, and after the title and abstract screening, we evaluated 125 full-text reports. Ultimately, 15 reports were included.

#### Characteristics of publications

Among the included reports, seven of the studies were from before 2020 and eight from after 2020, as displayed in Table 2. Nine studies were from North America (USA [53–57] and Canada [58–61], three were from Europe (Denmark [62], Italy [63] and Germany and the Czech Republic [64]), one was from Asia (Iran [65]), and the remaining two were from Australia [66, 67]. The studies included a wide variety of methods, from observational studies [54, 63] to RCTs [54, 58], and a range of pilot [53, 60–62], acceptability [57], feasibility [56] and usability studies [65].

**Table 2:** Characteristics of the included reports (*N* = 15)

Region	Author, Year	Design	Type of data	Sample	Aim	Technology	Ethical	Legal	Social
Australia	Bradford, Armfield et al. 2014 [66]	Cost-minimisation analysis	Quantitative	Children in PPC *N* = 95 (video-consultations)	Comparison of costs of home vs. video consultation	Remote video consultation	Reduce burdens; increase trust;	Equal access;	Cost saving
Australia	Bradford, Young et al. 2014 [67]	Qualitative, semi-structured interviews	Qualitative	Palliative care clinicians *N* = 10	Investigation of home telehealth from clinicians’ viewpoints	Remote video consultation	Obstacle to quality of care; increase trust; increased autonomy/agency; empowerment; contact;	Privacy; equal access;	Cost saving & training; changing relations; communication issues
North America	Breakey et al. 2022 [58]	RCT	Quantitative	Adolescents with cancer Age: 12–18 years) *N* = 81	Evaluation of a self-management programme	Web-based	Increased burdens;	Participation in decisions	
North America	Fortier et al. 2016 [53]	Pilot study	Quantitative	Adolescents with cancer Age: 8–18 years) *N* = 12	Evaluation of pain management	App	Obstacle to quality of care; engaging	Equal access;	Cost saving; smartphones
Europe	Hoffmann et al. 2021 [64]	Qualitative study; vignette usage scenarios	Qualitative	Adolescents with leukaemia or solid cancers Age: 6–17 years) *N* = 58	Investigating the design process	App	Engaging;	Participation in decisions; standardisation	Smartphones
North America	Hunter et al. 2020 [54]	Prospective, observational cohort study/RCT	Quantitative	Adolescents undergoing cancer treatment Age: 8–18 years Intervention *N* = 20 Control *N* = 28	Efficacy of a pain management tool	App	Reduce harm; reduce burden; increase quality of care		Cost saving; smartphones
North America	Jibb et al. 2023 [55]	Parent co-design approach	Mixed Methods	Parents of children 2–11 years) with cancer *N* = 22	Testing of a pain management tool	App	Engaging	Participation in decisions;	Changing relations
North America	Jibb et al. 2017 [59]	One-group pre/poststudy design	Quantitative	Adolescents with cancer Age; 12–18 years *N* = 40	Evaluation of a pain management tool prior to an RCT	App	Reduce harm; increase QoL; engaging; timely access to care	Participation in decisions;	
North America	Jibb et al. 2018 [60]	Qualitative post pilot	Qualitative	Adolescents with cancer Age: 12–18 years *N* = 20	Evaluation of a pain management tool	App	Increased burdens; increased autonomy/agency;	Equal access; participation in decisions;	Cost saving
North America	Lai et al. 2015 [56]	Feasibility study	Quantitative	Patients with a cancer diagnosis Age: 7–21 years, *N* = 57 (and 13 clinicians)	Evaluation of a symptom monitoring and reporting tool	Web-based programme	Reduce burdens; information as burden;	Equal access; participation in decisions;	
Asia	Mehdizadeh et al. 2023 [65]	Usability study	Quantitative	Adolescents with cancer and their parents Age: 7–14 Children *N* = 19 Parents *N* = 25	Evaluation of a self-management tool	App	Empowerment;		Smartphones
North America	O’Sullivan et al. 2018 [61]	Qualitative pilot study	Qualitative	Children with cancer Age: 8–18 *N* = 20	Evaluation of a symptom screening tool	App	increase trust; engaging; participation;	Privacy	Cost saving & maintenance; smartphones
Europe	Tiozzo et al. 2021 [63]	Observational prospective study	Quantitative	Children with hematologic or solid tumours Age: 4–21 *N* = 124	Evaluation of a pain management tool	App	Improved quality of care; empowerment		
North America	Weaver, Robinson, et al. 2020 [57]	Case series.	Quantitative	Children in home hospice for end-of-life care + Caregivers + HCP Children *N* = 15 Caregivers *N* = 13 HCP = 15	Acceptability of telehealth in homes	Remote video consultation	increased autonomy/agency; empowerment; participation; increased burdens	Equal access; participation in decisions;	Cost saving; changing relations
Europe	Weibel et al. 2020 [62]	Qualitative pilot study	Qualitative	Children with cancer and their social connections Age: 12–14 Children *N* = 3 Parent *N* = 3 Teacher *N* = 2 Peers *N* = 15	Exploration of social and academic connections through an avatar presence in schools	Robot/avatar	increased autonomy/agency; inclusion; exclusion; empowerment; contact; increased burdens	Equal access;	Cost saving & training

## Results of synthesis

### Ethical aspects

Several technologies were reported to have the potential to *avoid or reduce harm* (e.g. health technologies, including self-management tools, can reduce severe to moderate pain [54, 59]), and the newer formats that facilitate contact with HCPs, such as video apps, may reduce burdens such as the time spent on visits, travelling, reporting, and completing forms [54, 56, 66]. These activities can constitute health hazards and psychological burdens for parents and children. Moreover, home-based multipurpose technologies can prevent gaps or lags in children’s education [62].

In terms of *benefits*, in two studies, HCPs received constant reports on changes in patients’ symptoms through a monitoring app that could provide timely responses [63], which increased QoL [59] and quality of care [54]. However, interventions and follow-up for patients depended on being able to contact them; hence, such follow-up must not rely entirely on the app, but should be complemented with phone or other means of communication [59]. Furthermore, this constant reporting could be experienced as a burden by children who prefer face-to-face communication with HCPs when they feel sick [60].

There were several obstacles to realising the benefits of video communication, with HCPs expressing uncertainty about the quality of care. They cited challenges such as the loss of personal contact, the absence of non-verbal and visual cues, and technology failures at critical deficiencies or vulnerabilities [53, 67].

Continuity of contact with dedicated HCP teams in home settings was valued because it provided continuity for whole families as well as HCPs and consequently increased trust when done well [61, 66, 67]. However, not all regular contact was universally experienced as helpful, such as in the case of parents who received weekly fatigue symptom reports that were meant as facilitating for dialogue with HCPs, even though most appreciated these reports [56].

Some reports touched upon what digital solutions contribute or add to interactions among children, their families, and HCPs in terms of *agency and autonomy* [57, 60, 62, 67]. Some aspects of the technologies that affected children’s agency were identified in the reports [53, 55, 59, 61, 62, 64]. For many children, solutions that required regular reading sessions in a manner similar to school were considered unhelpful [58]. Apps developed with children [53, 55, 59, 61], and gamified content [64] that comprised interaction and age-appropriate design, were highlighted as means to engage children in their own situations and experiences while providing symptom information to HCPs, children, and caregivers. A telepresence robot (i.e. an audio-video device shaped like a face) fostered children’s feelings of inclusion and participation while maintaining their educational advancement [62]. However, the use of telepresence robots for informal school activities outside the classroom or during movements between classrooms when the robot had to be carried gave rise to feelings of dependence. Although robots could facilitate care, the disembodied experience seemed to be underlined by such situations [62].

A theme identified in several of the reports was an existential understanding of *empowerment* [57, 62, 67] – in the sense that the children can create a meaningful future [68]. The ability to participate in social and educational activities seems to create a sense of normality for children [57]. Furthermore, access and frequent contact give children and their caregivers a fundamental feeling of being remembered and knowing what decisions are being made [62, 67]. When children are allowed to self-report and engage in their own situations through adapted designs and questions developed through user participation [61], they may experience less serious pain [54, 59] and obtain more timely access to care [59, 63], but with little direct effect on self-efficacy [59]. The design of apps and the formulation of questions can be welcoming and provide a sense of empowerment for children and parents [65]. However, if the quality of the apps is mainly tested through time spent on the apps and adherence to digital solutions [65], the sense of empowerment may be instrumentalised for HCPs’ purposes. Furthermore, reporting can be experienced as overwhelming or structured for the HCPs’ sakes [57, 58, 60, 62].

### Legal aspects

One of the central aspects of autonomy is *privacy*, understood as the right to control who has access to and can use personal information about a child—families or HCPs [69]. One central new issue with apps is the storage of information and its transmission between children and their HCPs [61, 67]. In terms of privacy, there is a difference between self-reporting though apps and video conferences in the home. In the first case, other than being asynchronous, the child (or the parent) has improved control over who has access, which enhances autonomy. Being able to control who knows something about a person is also a way to control who influences that person’s actions. In the second case, it is more challenging to have such control [67]. In addition, during video conferences, it can be difficult to know who is visiting who and, consequently, whether the patient or the HCP should steer the session [67].

Several studies have emphasised that the transfer to health technologies for PPC enhanced both access to services and the continuity of such services, which increased equity [53, 57, 66, 67]. However, being given access to the same service does not mean that different children and families receive *just* or *fair* care since families also need access to digital infrastructure [67]. Children and families have different needs and preferences regarding how and when apps report back to them [56, 60]. When linking schools to these care services, the competencies of the staff and the social environment become central factors in determining how equitable the services are [62].

A different aspect of justice underlying some of the reports was children’s and families’ participation in the planning and delivery of care or services through apps [56–58, 64]. A central component of such participation is prospective users’ or user groups’ involvement in the design and development of apps [55, 59, 60].

One report discussed the importance of aiming for a *conformité européenne* (CE) mark as a seal of approval for medical devices and developed apps [64]. However, we found no similar discussions in the other reports.

### Social aspects

The included reports listed *beneficence* among the economic benefits of health systems for families in terms of time, money, and resources [53, 54, 57, 60, 66]. They also highlighted the costs connected to video consultation equipment and its development and maintenance [66]. The training and good practices of HCPs and other professionals who deal with children are prerequisites for realising the benefits discussed above [62, 67]. However, apps with clear health scopes need to be aligned with health systems, and new costs might be incurred in maintaining and updating these systems [61].

To some extent, health technologies have transformed the relationships of parents and/or caregivers of children in PPC with children [67]. Registration of a child’s pain on an app could translate into advice strategies and provide connections to health services [55]. In addition, anticipatory pain management can change, adjust, and calibrate expectations of future health and QoL [57].

Through the apps, parents can gain access to their children’s own health assessments, but the design and delivery modes for such information are critical. One report documented that 58% of parents found their children’s own assessments of fatigue helpful, but only 38% found them helpful in discussions with HCPs, even though the technical solutions were reported to be user-friendly [56].

For HCPs, video consultations can be challenging because they provide a limited perspective, relying mainly on sound and vision, while lacking input from other senses. This limitation may cause particular difficulties when communicating across cultural, linguistic, or socioeconomic divides [67]. This difficulty suggested the need for HCPs to develop compensatory strategies when care delivery becomes difficult and caring situations demand some reconfiguration, such as with video conversations in which HCPs found it valuable to see children’s full social settings. However, this situation again created ambivalence regarding whether the HCPs were in their clinics or visiting [67]. Furthermore, HCPs reported that the switch from phone to video caused inflexibility: phone call follow-ups with patients could be done in the car or between appointments, while the fixed scheduling of video conferences reduced flexibility [67].

No reports discussed directly at what age and under what circumstances children should have their own smartphones. The reports assumed apps and smartphones to be available to 4–21-year-olds [63], 6–17-year-olds [64], 8–18-year-olds [53, 54, 61], 12–18-year-olds [59], and from 7 years onward [65].

## Discussion

### Principal findings

The overall aim of this systematic review was to analyse the ELSA of health technology for home-based PPC. Some findings were unsurprising and are receiving due consideration in research, policy, and practice discussions about health technologies for home-based PPC. These findings will be briefly summarised before we address lacunas and issues where more urgent attention is required.

The studies on health technologies for home-based PPCs is dominated by socioethical issues regarding how effectively health apps and solutions co-developed with children and their parents enhance benefits, autonomy, and access to services. Furthermore co-development activities foster empowerment over people’s personal situations. Moreover, the ethical and legal issue of equitable access to services is a strong driver for moving health technologies into homes. Attention has also been paid to ethical and legal risks to autonomy because privacy is affected when new data layers come between patients and HCPs.

In the following sections, we will further discuss the ambivalent socioethical aspects and the potential transformations that may occur when whole families are included in video communication as opposed to apps that only target sick children. The socioethical issue of the age at which children should be introduced to smartphones was largely ignored in the reviewed reports, and there are increasing ethical, legal, and social issues related to the use of apps as health technologies.

#### The transformative nature of technologies

One issue in the findings that merits further enquiry is how home-based health technologies transform relationships between children and their families and HCPs. These relationships based on technologies and agency for children should be examined further. In a review conducted by Schröder et al. [7], HCPs reported that digital communication changed their work and relationships with care recipients. Verkerk et al. [34, 35] discussed an ‘ethics of families’, where the locus of value is in the relationships between family members. This locus is based on the idea that changes in relationships due to increased family participation through video conferencing, and reduced family inclusion through apps, may have socioethical effects. Furthermore, as documented regarding health technologies for PPC and elsewhere, there are discrepancies between patient and proxy reporting [70–72]. Since such issues did not seem to influence the validated instruments used in the reviewed studies, such as pain scales [59] or QoL [64], the triangle of self-reporting, change of agency, and family relationships should be further researched.

#### Introducing smartphones for PPC

A central finding was that none of the included reports discussed directly at what age and under what circumstances children in palliative care should have their own smartphones. Several research projects have used smartphones as the delivery mechanism for services, but regarding the issue of when children should acquire smartphones, there is a debate about the possibility of smartphones being obstacles to well-being [37]. The effects of smartphones on children may be connected to their social backgrounds [73]. In the reviewed reports, empowerment and agency as well as participation in one’s own health and social settings are highlighted when analysing apps [55–65, 67]. What might raise concern is that smartphone usage comes with new uncertainties. Children with sound networks and good relationships with their parents are less at risk of smartphone addiction [74]. Social media might have positive networking effects for adolescents and young adults [75]. However, while using social media to interact with peers can prevent feelings of isolation, smartphones may make socially isolated children feel even more isolated [74]. A relevant issue is that several countries are considering setting age limits for the use of social media. Recently, Australia banned social media for children under the age of 16 [76].

Regarding the issue of introducing smartphones to children, it seems likely that value conflicts will occur [41]. It is unlikely that any of the current ethical principles regarding PPC (the best-interest, risk–benefit proportionality, distributive justice, and autonomy principles [2]) can directly resolve such conflicts. One aspect to consider in introducing smartphones is children’s digital literacy rather than their technological mastery and aptness, since young users may be more naïve than adults [77] Other aspects that might be considered are the parents’ or families’ views and values regarding their children’s digital lives. Furthermore, if access to care services depends on smartphones, then health services demand that children provide personal data to the smartphone industry. Consequently, future researchers should pay attention to the role health services should play in introducing smartphones into the lives of children in PPC, and discussions should be raised on this matter in clinical practice.

#### ELSA issues with apps

Surprisingly, only two of the included reports discussed privacy directly. Seen from the ELSA perspective, this discussion of privacy, especially concerning apps, could be further developed regarding two different strands of research. The first strand could involve reflections on the meaning of privacy in actual cases, which aspects of privacy are most at risk [69], and whether tools to mitigate privacy risks are suitable for these cases [78]. A second strand could involve reflections on current data-sharing laws and regulations (especially in the case of rare diseases) [16]. Thorogood [79] highlighted that ‘many patients with rare diseases see the important clinical and scientific value of data sharing’ (p. 6) [79] and concluded by stressing the importance of finding legal ways to facilitate international cooperation and data sharing in an age of increasingly strict data governance legislation. Recently, suggestions have been made that governments should actively support data solidarity [80].

A different ELSA issue is that health technology devices, apps, and solutions should be given formal status. Only one of the included reports discussed the value of aiming for a CE mark as a seal of approval for a medical device or developed app. This was surprising since the approval of medical devices according to standards connects to the possibility of including them in health systems and the overall health economy [81, 82]. Hofmann et al. [64] stated that they had chosen ‘to avoid claiming a CE mark for the respective software platform as if it was a medical device, [although] we acknowledge the value of such a process and we identify it as a key potential future step’. In developing their solution, the authors followed the European AI Guidelines, which state that solutions must be lawful, ethical, and robust [32]. These guidelines define robustness as ‘resilience to attack and security, fall back plan and general safety, accuracy, reliability, and reproducibility’ [83]. For purchasers, a product without CE approval as a medical device could easily be perceived as lacking one or several of these properties. The European Union (EU) AI Act states that medical devices aiming to make decisions for different levels of medical aid should be classified as ‘high risk’ [84, 85], whereas monitoring apps that suggest exercise or diet should be classified as ‘low risk’ [85]. In this context, apps to support self-management that delegate some autonomy to the apps might be required to adhere to the requirements set out for high-risk use. Consequently, there are two important ELSA challenges to consider for further research:

1. At the outset of the development of such self-management apps, the legal framework must be well known, and the suggested app should be located within this framework.
2. These ambitions should be proportional to the burdens placed on the participants in the development (i.e. adhere to the risk–benefit principle discussed in the PPC literature [2, 86]).

Furthermore, independent studies will be needed on health technologies for home-based PPC, as well as on health technologies in general, regarding the relationship between their intended and actual use [87]. As Weaver et al. [88] claimed, the notions of burden and benefit are not well-defined (or understood) for participants in research in this category. Weaver et al.’s [88] point raises the issue of informed consent in this type of research, since having a shared notion of benefits and burdens between researchers and participants is a basic building block of mutual trust, communication, and understanding [89]. However, it is important that children receiving PPC be given opportunities to participate in research [42], and that healthcare personnel acknowledge the children’s right to decide for themselves, when they can to ensure relevant and timely research to the best of future digital health services.

### Strengths and limitations

ELSA of home-based PPC should be considered in light of broad health systems and modelled accordingly [90]. Understanding ELSA within this context, as suggested by Boyden et al. [91], would constitute an important contribution to the home technologies for PPC. A strength of the current review was that the review protocol was registered in PROSPERO a priori and was performed in line with acknowledged methodological guidance. The search strategy was developed in collaboration with research librarians, and pairs of authors independently assessed the eligibility of the studies and extracted data. Furthermore, we limited the included reports to peer-reviewed studies and did not include grey literature or conference proceedings. A strength of this approach was that the findings were reliable, although potentially at the cost of breadth and comprehensiveness. However, it is likely that issues discussed in the grey literature were also addressed in peer-reviewed articles, but maybe in more detail in the grey literature. Since there is no general approach to ELSA studies and literature reviews, the structuring of the findings was largely at the discretion of the authors. However, as an interdisciplinary team, we used our collaborative capacities to discuss emerging issues based on the findings.

A limitation of the review may be that we were unable to identify all the relevant search terms for health technologies and PPC. Furthermore, the inclusion criteria had some language restrictions. Therefore, some reports may have been missed.

## Conclusion

The central ethical issues found above are co-development activities that strengthen all involved parties and create a solid foundation for improved home-based PPC for children and their families that strengthens agency and empowerment. Seen from a legal perspective, such activities support the right to be active in and have control over one’s life. The social issues concerning health technologies for PPC are connected to health economics on the one hand and internal family dynamics on the other.

Despite the preoccupation with privacy, there is a dilemma in this field between the need to protect health information and keep it secret and the need for data sharing though health technologies for home-based PPC. The collection and processing of health data are closely connected to wider health systems, and this connection depends on strong quality control and the formal approval of medical technology. However, there are some blind spots in this respect, such as the seemingly naïve enthusiasm for apps and smartphones. Thus, continuous discussion on the ethical, legal and social issues arising from technology in PPC should be pursued in clinical settings. Quality of care, and children’s and parents’ access to services and participation in planning care, are high on the agenda. Seen from an ELSA perspective, the implications for further research fall into three areas: the introduction of smartphones to support PPC, the sociolegal requirements for apps used for PPC, and the transformative character of health technologies.

## Electronic supplementary material

Below is the link to the electronic supplementary material.

Supplementary Material 1: Reporting checklist.

Supplementary Material 2: Deviations from the protocol.

Supplementary Material 3: Complete overview of search strategies.

## Data Availability

Data are available upon reasonable request to the corresponding author.

## References

[1] Winger A, Kvarme LG, Løyland B, Kristiansen C, Helseth S, Ravn IH. Family experiences with palliative care for children at home: a systematic literature review. BMC Palliat Care. 2020;19(1):165. 33099303 10.1186/s12904-020-00672-4 PMC7585197

[2] Benini F, Papadatou D, Bernadá M, Craig F, De Zen L, Downing J, et al. International standards for pediatric palliative care: from impacct to GO-PPaCS. J Pain Symptom Manage. 2022;63(5):e529–43. 35031506 10.1016/j.jpainsymman.2021.12.031

[3] Kassam A, Skiadaresis J, Alexander S, Wolfe J. Parent and clinician preferences for location of end-of-life care: home, hospital or freestanding hospice? Pediatr Blood Cancer. 2014;61(5):859–64. 24265171 10.1002/pbc.24872

[4] Bradford N, Armfield NR, Young J, Smith AC. The case for home based telehealth in pediatric palliative care: a systematic review. BMC Palliat Care. 2013;12(1):4. 23374676 10.1186/1472-684X-12-4 PMC3584741

[5] Bird M, Li L, Ouellette C, Hopkins K, McGillion MH, Carter N. Use of synchronous digital health technologies for the care of children with special health care needs and their families: scoping review. JMIR Pediatr Parent. 2019;2(2):e15106. 31750840 10.2196/15106 PMC6895870

[6] Thorstensen E. Responsible assessments. Frameworks for a Value-Based governance of assistive technologies. [PhD]. [Oslo]: OsloMet. Centre for the Study of Professions; 2020.

[7] Schröder J, Riiser K, Holmen H. Healthcare personnel’s perspectives on health technology in home-based pediatric palliative care: a qualitative study. BMC Palliat Care. 2024;23(1):137. 38811957 10.1186/s12904-024-01464-w PMC11134737

[8] Holmen H, Riiser K, Winger A. Home-Based pediatric palliative care and electronic health: systematic mixed methods review. J Med Internet Res. 2020;22(2):e16248. 32130127 10.2196/16248 PMC7070344

[9] Miller KA, Baird J, Lira J, Herrera Eguizabal J, Fei S, Kysh L, et al. The use of telemedicine for Home-Based palliative care for children with serious illness: A scoping review. J Pain Symptom Manag. 2021;62(3):619–e6366. 10.1016/j.jpainsymman.2020.12.004 33348029

[10] Hofmann B. Biases and imperatives in handling medical technology. Health Policy Technol. 2019;8(4):377–85.

[11] Lehoux P, Blume S. Technology assessment and the sociopolitics of health technologies. J Health Polit Policy Law. 2000;25(6):1083–120. 11142053 10.1215/03616878-25-6-1083

[12] May C, Mort M, Williams T, Mair F, Gask L. Health technology assessment in its local contexts: studies of telehealthcare. Soc Sci Med. 2003;57(4):697–710. 12821017 10.1016/s0277-9536(02)00419-7

[13] Mort M, May CR, Williams T. Remote Doctors and Absent Patients: Acting at a Distance in Telemedicine? Science, Technology, & Human Values. 2003;28(2):274–95.

[14] van der Wilt GJ, Reuzel R, Banta HD. The ethics of assessing health technologies. Theor Med Bioeth. 2000;21(1):101–13. 10.1023/a:1009934700930 10927971

[15] Aicardi C, Reinsborough M, Rose N. The integrated ethics and society programme of the human brain project: reflecting on an ongoing experience. J Responsible Innov. 2018;5(1):13–37.

[16] Alvarez MJR, Griessler E, Starkbaum J, Ethical. Legal and Social Aspects of Precision Medicine. In: Hasanzad M, editor. Precision Medicine in Clinical Practice [Internet]. Singapore: Springer Nature; 2022 [cited 2023 Aug 22]. pp. 179–96. Available from: 10.1007/978-981-19-5082-7_11

[17] Zwart H, Landeweerd L, van Rooij A. Adapt or perish? Assessing the recent shift in the European research funding arena from ‘ELSA’ to ‘RRI’. Life Sci Soc Policy. 2014;10(1):1–19. 26085447 10.1186/s40504-014-0011-x PMC4648839

[18] Zwart H, Nelis A. What is ELSA genomics? EMBO Rep. 2009;10(6):540–4. 19488040 10.1038/embor.2009.115 PMC2711829

[19] Delgado RodríguezJ, Ramos-García V, Infante‐Ventura D, Suarez‐Herrera JC, Rueda‐Domínguez A, Serrano‐Aguilar P, et al. Ethical, legal, organizational and social issues related to the use of scalp cooling for the prevention of chemotherapy‐induced alopecia: A systematic review. Health Expect. 2022;26(2):567–78. 36585793 10.1111/hex.13679 PMC10010082

[20] Müller R, Klemmt M, Ehni HJ, Henking T, Kuhnmünch A, Preiser C, et al. Ethical, legal, and social aspects of symptom checker applications: a scoping review. Med Health Care Philos. 2022;25(4):737–55. 36181620 10.1007/s11019-022-10114-y PMC9613552

[21] Čartolovni A, Tomičić A, Lazić Mosler E. Ethical, legal, and social considerations of AI-based medical decision-support tools: A scoping review. Int J Med Informatics. 2022;161:104738. 10.1016/j.ijmedinf.2022.104738 35299098

[22] Heggestad AKT, Magelssen M, Pedersen R, Gjerberg E. Ethical challenges in home-based care: A systematic literature review. Nurs Ethics. 2021;28(5):628–44. 33334250 10.1177/0969733020968859

[23] Kaplan B, Ethical. Legal, and Social Issues Pertaining to Virtual and Digital Representations of Patients. In: Hsueh PYS, Wetter T, Zhu X, editors. Personal Health Informatics: Patient Participation in Precision Health [Internet]. Cham: Springer International Publishing; 2022 [cited 2024 May 29]. pp. 519–42. Available from: 10.1007/978-3-031-07696-1_23

[24] Steen M. ELSA-in-a-box; a framework to integrate ethical, legal, and societal aspects in the development and deployment of AI systems [Internet]. Erasmus University Rotterdam. 2023 [cited 2024 May 30]. Available from: https://www.eur.nl/en/news/elsa-box-framework-integrate-ethical-legal-and-societal-aspects-development-and-deployment-ai

[25] Thorstensen E. Literature review of responsible research and innovation on assisted living technologies for the Assisted Living Project [Internet]. Oslo and Akershus University College of Applied Sciences; 2017. Available from: https://www.researchgate.net/publication/316541124_Literature_review_of_responsible_research_and_innovation_on_assisted_living_technologies_for_the_Assisted_Living_Project?channel=doi%26linkId=5902d70daca2725bd722476c%26showFulltext=true

[26] Hofmann B. Ethical challenges with welfare technology: A review of the literature. Sci Eng Ethics. 2013;19(2):389–406. 22218998 10.1007/s11948-011-9348-1

[27] Felber NA, Tian YJ (Angelina), Pageau F, Elger BS, Wangmo T, editors. Mapping ethical issues in the use of smart home health technologies to care for older persons: a systematic review. BMC Med Ethics. 2023;24(1):24. 10.1186/s12910-023-00898-w PMC10061702 36991423

[28] Mort M, Roberts C, Pols J, Domenech M, Moser I. Ethical implications of home Telecare for older people: a framework derived from a multisited participative study. Health Expect. 2015;18(3):438–49. 23914810 10.1111/hex.12109 PMC5060789

[29] Pols J. Telecare. What patients care about. In: Mol A, Moser I, Pols J, editors. Care in practice: on tinkering in clinics, homes and farms. transcript; 2010. pp. 171–94.

[30] Demiris G, Oliver DRP, Fleming DA, Edison K. Hospice staff attitudes towards telehospice. Am J Hosp Palliat Care. 2004;21(5):343–7. 15510570 10.1177/104990910402100507

[31] Demiris G, Oliver DP, Courtney KL. Ethical considerations for the utilization of tele-health technologies in home and hospice care by the nursing profession. Nurs Adm Q. 2006;30(1):56–66. 16449885 10.1097/00006216-200601000-00009

[32] Garani-Papadatos T, Natsiavas P, Meyerheim M, Hoffmann S, Karamanidou C, Payne SA. Ethical principles in digital palliative care for children: the MyPal project and experiences made in designing a trustworthy approach. Front Digit Health. 2022;4:730430. 35373180 10.3389/fdgth.2022.730430 PMC8971573

[33] Steindal SA, Klarare A, Sørensen BS, Holmen H, Nes AAG, Winger A et al. Ethical Considerations Regarding Digital Health Services in Home-Based Palliative Care: A Subanalysis of 2 Reviews. Journal of Hospice & Palliative Nursing. 2024;10.1097/NJH.0000000000001072. 10.1097/NJH.0000000000001072 39774043

[34] Verkerk MA. Families and End of Life Care. In: Emmerich N, Mallia P, Gordijn B, Pistoia F, editors. Contemporary European Perspectives on the Ethics of End of Life Care [Internet]. Cham: Springer International Publishing; 2020 [cited 2025 Feb 4]. pp. 355–66. Available from: 10.1007/978-3-030-40033-0_23

[35] Verkerk MA, Lindemann H, McLaughlin J, Scully JL, Kihlbom U, Nelson J, et al. Where families and healthcare Meet. J Med Ethics. 2015;41(2):183–5. 25210197 10.1136/medethics-2013-101783

[36] Christakis DA, Hale L, editors. Handbook of Children and Screens: Digital Media, Development, and Well-Being from Birth Through Adolescence. 1st ed. 2025. Cham: Springer Nature Switzerland; 2025. 1 p.

[37] Haidt J. The anxious generation: how the great rewiring of childhood is causing an epidemic of mental illness. New York: Penguin; 2024. p. 400.

[38] Gkintoni E, Vantaraki F, Skoulidi C, Anastassopoulos P, Vantarakis A. Promoting physical and mental health among children and adolescents via Gamification—A. Concept Syst Rev Behav Sci. 2024;14(2):102. 10.3390/bs14020102 PMC10886329 38392455

[39] Prinsloo P, Dada S, Bastable K, Raghavendra P, Granlund M. The application of the family of participation-related constructs (fPRC) framework to AAC intervention outcomes in children with complex communication needs: a scoping review. Augmentative Altern Communication. 2024;40(3):182–95. 10.1080/07434618.2023.2276701 37994791

[40] Foster CC, Morales L, Fawcett AJ, Coleman CL. Access and quality of pediatric home healthcare: A systematic review. Home Health Care Manage Pract. 2023;35(4):287–98.

[41] Schofield G, Dittborn M, Huxtable R, Brangan E, Selman LE. Real-world ethics in palliative care: A systematic review of the ethical challenges reported by specialist palliative care practitioners in their clinical practice. Palliat Med. 2021;35(2):315–34. 33302783 10.1177/0269216320974277 PMC7897798

[42] United Nations. Convention on the Rights of the Child [Internet]. General Assembly resolution 44/25 11, 1989. Available from: https://www.ohchr.org/en/instruments-mechanisms/instruments/convention-rights-child

[43] McCullough LB, Coverdale JH, Chervenak FA. Constructing a systematic review for Argument-Based clinical ethics literature: the example of concealed medications. J Med Philosophy: Forum Bioeth Philos Med. 2007;32(1):65–76. 10.1080/03605310601152206 17365446

[44] Page MJ, McKenzie JE, Bossuyt PM, Boutron I, Hoffmann TC, Mulrow CD, et al. The PRISMA 2020 statement: an updated guideline for reporting systematic reviews. BMJ. 2021;372:n71. 33782057 10.1136/bmj.n71 PMC8005924

[45] Lockwood C, Porritt K, Munn Z, Salmond S, Bjerrum M, Loveday H et al. Systematic reviews of qualitative evidence. In: Aromataris E, Lockwood C, Porritt K, Pilla B, Jordan Z, editors. JBI Manual for Evidence Synthesis [Internet]. JBI; 2024. Available from: https://synthesismanual.jbi.global

[46] Veritas Health Innovation. Covidence systematic [internet]eview software [Internet]. Melbourne, Australia: Veritas Health Innovation; Available from: www.covidence.org.

[47] Kahrass H, Borry P, Gastmans C, Ives J, van der Graaf R, Strech D et al. PRISMA-Ethics – Reporting Guideline for Systematic Reviews on Ethics Literature: development, explanations and examples [Internet]. OSF Preprints; 2021 [cited 2023 Sep 27]. Available from: https://osf.io/g5kfb/

[48] Hofmann B, Droste S, Oortwijn W, Cleemput I, Sacchini D. Harmonization of ethics in health technology assessment: A revision of the socratic approach. Int J Technol Assess Health Care; Camb. 2014;30(1):3–9. 10.1017/S0266462313000688 24499630

[49] McDougall RJ, Notini L. Overriding parents’ medical decisions for their children: a systematic review of normative literature. J Med Ethics. 2014;40(7):448–52. 23824967 10.1136/medethics-2013-101446

[50] Wieczorek M, O’Brolchain F, Saghai Y, Gordijn B. The ethics of self-tracking. A comprehensive review of the literature. Ethics Behav. 2023;33(4):239–71.

[51] Tomičić A, Malešević A, Čartolovni A. Ethical, legal and social issues of digital phenotyping as a future solution for Present-Day challenges: A scoping review. Sci Eng Ethics. 2021;28(1):1. 34928438 10.1007/s11948-021-00354-1 PMC8686352

[52] Thomas J, Harden A. Methods for the thematic synthesis of qualitative research in systematic reviews. BMC Med Res Methodol. 2008;8(1):45. 18616818 10.1186/1471-2288-8-45 PMC2478656

[53] Fortier MA, Chung WW, Martinez A, Gago-Masague S, Sender L. Pain Buddy: A novel use of m-health in the management of children’s cancer pain. Computers Biology Med. 2016;76:202–14. 10.1016/j.compbiomed.2016.07.012 PMC5639256 27479493

[54] Hunter JF, Acevedo AM, Gago-Masague S, Kain A, Yun C, Torno L, et al. A pilot study of the preliminary efficacy of pain Buddy: A novel intervention for the management of children’s cancer‐related pain. Pediatr Blood Cancer. 2020;67(10):e28278. 32743950 10.1002/pbc.28278 PMC9977267

[55] Jibb LA, Liu W, Stinson JN, Nathan PC, Chartrand J, Alberts NM et al. Supporting parent capacity to manage pain in young children with cancer at home: Co-design and usability testing of the PainCaRe app. Paediatric and Neonatal Pain [Internet]. 2023; Available from: https://onlinelibrary.wiley.com/journal/26373807 10.1002/pne2.12097 PMC11514300 39473834

[56] Lai JS, Yount S, Beaumont JL, Cella D, Toia J, Goldman S. A patient-centered symptom monitoring and reporting system for children and young adults with cancer (SyMon-SAYS): SyMon-SAYS. Pediatr Blood Cancer. 2015;62(10):1813–8. 25856587 10.1002/pbc.25550

[57] Weaver MS, Robinson JE, Shostrom VK, Hinds PS. Telehealth acceptability for children, family, and adult hospice nurses when integrating the pediatric palliative inpatient provider during sequential rural home hospice visits. J Palliat Med. 2020;23(5):641–9. 31808722 10.1089/jpm.2019.0450

[58] Breakey VR, Gupta A, Johnston DL, Portwine C, Laverdiere C, Le May S, et al. A pilot randomized control trial of teens taking charge: A web-based self-management program for adolescents with cancer. J Pediatr Hematol / Oncol Nurs. 2022;39(6):366–78. 35759365 10.1177/27527530211068778

[59] Jibb LA, Stevens BJ, Nathan PC, Seto E, Cafazzo JA, Johnston DL, et al. Implementation and preliminary effectiveness of a real-time pain management smartphone app for adolescents with cancer: A multicenter pilot clinical study. Pediatr Blood Cancer. 2017;64(10):e26554. 10.1002/pbc.26554 28423223

[60] Jibb LA, Stevens BJ, Nathan PC, Seto E, Cafazzo JA, Johnston DL, et al. Perceptions of adolescents with Cancer related to a pain management app and its evaluation: qualitative study nested within a multicenter pilot feasibility study. JMIR Mhealth Uhealth. 2018;6(4):e80. 29625951 10.2196/mhealth.9319 PMC5910537

[61] O’Sullivan C, Lee Dupuis L, Gibson P, Johnston DL, Baggott C, Portwine C, et al. Evaluation of the electronic self-report symptom screening in pediatrics tool (SSPedi). BMJ Support Palliat Care. 2018;8(1):110–6. 27803061 10.1136/bmjspcare-2015-001084

[62] Weibel M, Nielsen MKF, Topperzer MK, Hammer NM, Møller SW, Schmiegelow K, et al. Back to school with telepresence robot technology: A qualitative pilot study about how telepresence robots help school-aged children and adolescents with cancer to remain socially and academically connected with their school classes during treatment. Nurs Open. 2020;7(4):988–97. 32587717 10.1002/nop2.471 PMC7308694

[63] Tiozzo E, Fondi S, Biagioli V, Piccinelli E, Alibrandi F, Gawronski O, et al. Electronic assessment and tracking of pain at home: A prospective study in children with hematologic or solid tumors. J Pediatr Oncol Nurs. 2021;38(2):82–93. 33269620 10.1177/1043454220975443

[64] Hoffmann S, Schraut R, Kröll T, Scholz W, Belova T, Erhardt J, et al. AquaScouts: ePROs implemented as a serious game for children with Cancer to support palliative care. Front Digit Health. 2021;3:730948. 34957461 10.3389/fdgth.2021.730948 PMC8692290

[65] Mehdizadeh H, Asadi F, Nazemi E, Mehrvar A, Yazdanian A, Emami H. A mobile Self-Management app (CanSelfMan) for children with Cancer and their caregivers: usability and compatibility study. JMIR Pediatr Parent. 2023;6:e43867. 36995746 10.2196/43867 PMC10132021

[66] Bradford NK, Armfield NR, Young J, Smith AC. Paediatric palliative care by video consultation at home: a cost minimisation analysis. BMC Health Serv Res. 2014;14(1):328. 25069399 10.1186/1472-6963-14-328 PMC4127437

[67] Bradford NK, Young J, Armfield NR, Herbert A, Smith AC. Home telehealth and paediatric palliative care: clinician perceptions of what is stopping Us?? BMC Palliat Care. 2014;13(1):29. 24963287 10.1186/1472-684X-13-29 PMC4069094

[68] Odh I, Löfving M, Klaeson K. Existential challenges in young people living with a cancer diagnosis. Eur J Oncol Nurs. 2016;24:54–60. 27697277 10.1016/j.ejon.2016.08.005

[69] Solove D. A taxonomy of privacy. Univ Pa Law Rev. 2006;154(3):477–560.

[70] Weaver MS, Shostrom VK, Neumann ML, Robinson JE, Hinds PS. Homestead together: pediatric palliative care telehealth support for rural children with cancer during home-based end‐of‐life care. Pediatr Blood Cancer. 2021;68(4):e28921. 33522720 10.1002/pbc.28921

[71] Weaver MS, Hanna R, Hetzel S, Patterson K, Yuroff A, Sund S, et al. A prospective, crossover survey study of Child- and Proxy-Reported quality of life according to spinal muscular atrophy type and medical interventions. J Child Neurol. 2020;35(5):322–30. 32009500 10.1177/0883073819900463

[72] Aasen ERHV, Søvik ML, Størdal K, Lee A. Are we on the same page?? Exploring pediatric patients’ involvement with advance care planning. J Pain Symptom Manag. 2023;66(3):e353–63. 10.1016/j.jpainsymman.2023.04.003 37054956

[73] Rosič J, Schreurs L, Janicke-Bowles SH, Vandenbosch L. Trajectories of digital flourishing in adolescence: The predictive roles of developmental changes and digital divide factors. Child Development [Internet]. 2024 [cited 2024 May 13];n/a(n/a). Available from: https://onlinelibrary.wiley.com/doi/abs/10.1111/cdev.14101 10.1111/cdev.14101 38613364

[74] Ihm J. Social implications of children’s smartphone addiction: The role of support networks and social engagement. 2018 Jun 1 [cited 2024 Aug 22]; Available from: https://akjournals.com/view/journals/2006/7/2/article-p473.xml 10.1556/2006.7.2018.48 PMC6174576 29865865

[75] Peat G, Rodriguez A, Smith J. Social media use in adolescents and young adults with serious illnesses: an integrative review. BMJ Supportive Palliat Care. 2019;9(3):235–44. 10.1136/bmjspcare-2018-001646 30514717

[76] Sullivan H. Australia passes world-first law banning under-16s from social media despite safety concerns. The Guardian [Internet]. 2024 Nov 28 [cited 2025 Feb 4]; Available from: https://www.theguardian.com/media/2024/nov/28/australia-passes-world-first-law-banning-under-16s-from-social-media-despite-safety-concerns

[77] Kemp E, Trigg J, Beatty L, Christensen C, Dhillon HM, Maeder A, et al. Health literacy, digital health literacy and the implementation of digital health technologies in cancer care: the need for a strategic approach. Health Promotion J Australia. 2021;32(S1):104–14. 10.1002/hpja.387 32681656

[78] van Dijk N, Gellert R, Rommetveit K. A risk to a right? Beyond data protection risk assessments. Comput Law Secur Rev. 2016;32(2):286–306.

[79] Thorogood A. International Data Sharing and Rare Disease: The Importance of Ethics and Patient Involvement. In: Wu ZH, editor. Rare Diseases [Internet]. IntechOpen; 2020 [cited 2025 Jan 9]. Available from: https://www.intechopen.com/chapters/71101

[80] El-Sayed S, Kickbusch I, Prainsack B. Data solidarity: operationalising public value through a digital tool. Glob Public Health. 2025;20(1):2450403. 39789994 10.1080/17441692.2025.2450403

[81] Wickson F, Forsberg EM. Standardising responsibility?? The significance of interstitial spaces. Sci Eng Ethics. 2015;21(5):1159–80. 25344842 10.1007/s11948-014-9602-4

[82] Timmermans S, Epstein S. A world of standards but not a standard world: toward a sociology of standards and standardization. Ann Rev Sociol. 2010;36(1):69–89.

[83] European Commission. Shaping Europe’s digital future. 2019 [cited 2022 Aug 1]. Ethics guidelines for trustworthy AI| Shaping Europe’s digital future. Available from: https://digital-strategy.ec.europa.eu/en/library/ethics-guidelines-trustworthy-ai

[84] Directorate-General for Communication. European Commissions. 2024 [cited 2024 Oct 23]. AI Act enters into force - European Commission. Available from: https://commission.europa.eu/news/ai-act-enters-force-2024-08-01_en

[85] van Kolfschooten H, van Oirschot J. The EU artificial intelligence act (2024): implications for healthcare. Health Policy. 2024;149:105152. 39244818 10.1016/j.healthpol.2024.105152

[86] Rapoport A, Addressing Ethical Concerns Regarding Pediatric Palliative Care Research. Arch Pediatr Adolesc Med. 2009;163(8):688–91. 19652098 10.1001/archpedi.163.8.688

[87] Onitiu D, Wachter S, Mittelstadt B. How AI challenges the medical device regulation: patient safety, benefits, and intended uses. J Law Biosci. 2024;lsae007.

[88] Weaver MS, Mooney-Doyle K, Kelly KP, Montgomery K, Newman AR, Fortney CA, et al. The benefits and burdens of pediatric palliative care and End-of-Life research: A systematic review. J Palliat Med. 2019;22(8):915–26. 30835596 10.1089/jpm.2018.0483 PMC6755658

[89] O’Neill O. Some limits of informed consent. J Med Ethics. 2003;29(1):4–7. 12569185 10.1136/jme.29.1.4 PMC1733683

[90] Pacifico Silva H, Lehoux P, Miller FA, Denis JL. Introducing responsible innovation in health: a policy-oriented framework. Health Res Policy Sys. 2018;16(1):90. 10.1186/s12961-018-0362-5 PMC6131953 30200985

[91] Boyden JY, Hill DL, LaRagione G, Wolfe J, Feudtner C. Home-Based care for children with serious illness: ecological framework and research implications. Children. 2022;9(8):1115. 35892618 10.3390/children9081115 PMC9330186

