# Supplementary material for: Ethical, legal, and social aspects of health technologies for home-based paediatric palliative care – a systematic review
Source: BMC Palliat Care. 2025 May 16;24:139. doi: 10.1186/s12904-025-01774-7 (PMC12082856; doi:10.1186/s12904-025-01774-7)
Supplement: Supplementary file 1 — Supplementary Material 1: Reporting checklist. [file 12904_2025_1774_MOESM1_ESM.docx]

**Appendix 1 Search strategy Medline**

Database: Medline via Ovid Ovid MEDLINE(R) and Epub Ahead of Print, In-Process, In-Data-Review & Other Non-Indexed Citations and Daily <1946 to November 22, 2023>

Search date: 27.11.2023

| **#** | **Searches** |
| --- | --- |
| 1 | Palliative Care/ or "Hospice and Palliative Care Nursing"/ or Terminal Care/ or Palliative Medicine/ or exp Advance Care Planning/ or Terminally ill/ or Hospice care/ |
| 2 | palliat*.tw,kw,kf. |
| 3 | ((terminal* or end stage* or endstage* or advanced stage* or late stage*) adj3 (disease* or ill* or care* or caring or treatment* or period* or nurs* or patient*)).tw,kw,kf. |
| 4 | ((incurabl* or irreversibl*) adj ill*).tw,kw,kf. |
| 5 | (eol or "end of life" or dying).tw,kw,kf. |
| 6 | (advance*1 adj3 (plan*1 or planning or directive*)).tw,kw,kf. |
| 7 | hospice*.tw,kw,kf. |
| 8 | ((life limit* or life threatening) adj3 (disease* or condition* or illness*)).tw,kw,kf. |
| 9 | (advance* adj (disease* or illness*)).tw,kw,kf. |
| 10 | Neoplasms/ or Cerebral Palsy/ or Brain Injuries/ or Spinal Cord Injuries/ |
| 11 | (cancer* or neoplasm* or cerebral pals* or brain injur* or spinal cord injur*).tw,kw,kf. |
| 12 | or/1-11 |
| 13 | telemedicine/ or telepathology/ or teleradiology/ or telerehabilitation/ |
| 14 | remote sensing technology/ |
| 15 | videoconferencing/ or webcasts as topic/ or webcast/ |
| 16 | Telecommunications/ or Wireless Technology/ |
| 17 | telephone/ or answering services/ or cell phone/ or smartphone/ or text messaging/ |
| 18 | Mobile Applications/ or computers/ or microcomputers/ or computers, handheld/ or minicomputers/ or User-Computer Interface/ or Computer assisted instruction/ |
| 19 | Internet-Based Intervention/ |
| 20 | (((wearable or wireless or portable) adj2 (technolog* or electronic* or device*)) or (digital adj2 medicine) or (technolog* adj2 (remote or health)) or (remote adj2 care) or ((mobile or internet or electronic* or robot* or remote or virtual or wireless) adj2 (consultation* or app* or device* or rehab* or communicat* or team*))).tw,kw,kf. |
| 21 | (telecommunicat* or tele communicat* or teleconferenc* or tele conferenc* or app or apps or mobile based or short message* service* or sms or textmessag* or text messag* or texting or videoconferenc* or video conferenc* or webconferenc* or web conferenc* or webcast* or web cast* or webinar* or web based or web camera* or web application*).tw,kw,kf. |
| 22 | (((electronic or mobile or digital) adj health) or ((information or communicat*) adj technolog*) or "application of technolog*").tw,kw,kf. |
| 23 | (smartphone* or smart phone* or cellphone* or cell phone* or mobilephone* or mobile phone* or personal digital assistant* or palmpilot* or palm pilot* or smarthome* or smart home* or touchscreen* or touch screen* or high tech* or hightech*).tw,kw,kf. |
| 24 | (telemedicin* or tele medicin* or telehealth* or tele health* or telecare* or tele care* or telecari* or tele cari* or emedic* or e medic* or ehealth* or e health* or mhealth* or m health* or ehomecare* or e homecare* or e home care* or telehomecare or tele homecare or tele home or telenurs* or tele nurs* or teletherap* or tele therap* or telerehab* or tele rehab* or erehab* or e rehab* or teleconsultation* or tele consultation* or videoconsultation* or video consultation* or telemonitor* or tele monitor* or connected car*).tw,kw,kf. |
| 25 | ((internet based intervention* or (digital or online)) adj2 intervention*).tw,kw,kf. |
| 26 | or/13-25 |
| 27 | 12 and 26 |
| 28 | adolescent/ or puberty/ or child/ or child, preschool/ or infant/ or infant, newborn/ or infant, large for gestational age/ or infant, low birth weight/ or infant, small for gestational age/ or infant, very low birth weight/ or infant, extremely low birth weight/ or infant, postmature/ or infant, premature/ or infant, extremely premature/ |
| 29 | Pediatrics/ |
| 30 | Young Adult/ |
| 31 | (pediatric* or paediatric* or peadiatric* or neonatal* or neo natal* or neonate* or newborn* or new born* or infant* or baby or babies or toddler* or child* or kid or kids or girl or girls or boy or boys or minors or underage* or under age* or teen* or youth* or youngster* or adolescent* or adolescence or preadoles* or pre adoles* or juvenil* or puber* or pubescen* or pre puber* or prepuber* or prepubescen* or pre pubescen* or schoolchild* or preschool* or (young adj2 (adult* or man or men or woman or women or person* or people))).tw,kw,kf. |
