# Supplementary material for: Ethical, legal, and social aspects of health technologies for home-based paediatric palliative care – a systematic review
Source: BMC Palliat Care. 2025 May 16;24:139. doi: 10.1186/s12904-025-01774-7 (PMC12082856; doi:10.1186/s12904-025-01774-7)
Supplement: Supplementary file 3 — Supplementary Material 3: Complete overview of search strategies. [file 12904_2025_1774_MOESM3_ESM.docx]

**Supplementary file 3** Deviations from the published protocol

| **Approach described in the protocol** | **Description of the**  **deviation from protocol** |
| --- | --- |
| Methods  The proposed systematic review will employ the framework suggested by McCullough et al , as also suggested by Hofmann et al. (1), which comprises four stages 1. identify a focused question; 2. conduct a literature search using key terms relevant to the focused question; 3. assess the adequacy of the argument-based methods of the papers identified; and 4. identify conclusions drawn in each paper and whether they apply to the focused question. The proposed systematic review will be reported according to the PRISMA-Ethics (2). Potential deviations from the review protocol will be reported in the proposed review. | We modified point 3 to be “Assess the adequacy of the ethical issues of the reports identified”  We further report according to  Page, Matthew J., Joanne E. McKenzie, Patrick M. Bossuyt, Isabelle Boutron, Tammy C. Hoffmann, Cynthia D. Mulrow, Larissa Shamseer, Jennifer M. Tetzlaff, Elie A. Akl, Sue E. Brennan, et al. 2021. The PRISMA 2020 Statement: An Updated Guideline for Reporting Systematic Reviews. *BMJ* 372 (March). British Medical Journal Publishing Group:n71. doi:[10.1136/bmj.n71](https://doi.org/10.1136/bmj.n71). |
| Data extraction (selection and coding)  The research team will develop a data extraction form in Covidence to extract data from the included studies which will be piloted by the ET and HH on 2-5 publications. Six pairs of authors will extract data; one will extract data, while another will check data accuracy against the included studies. | As we ended up with a limited number of studies, it was not deemed necessary to include additional extractors to ET (now EB) and HH. However, we conducted the same structure with EB making the first data extraction before HH read through the same studies. |
| Strategy for data synthesis  A deductive approach will be used to thematically group the data from the included studies. The ethical, legal and social aspects will guide the analysis and provide a structure where data from the included papers will be placed within according to higher order categories. See Table 1 in Schofield et al 2021 for an example. Based on the data in each aspect we will inductively redefine and revise the name of the themes. The data will be analyzed by ET, BH and HH, while all authors will agree upon the final names of the themes and appraise the relevant arguments and findings under these themes. We will apply investigator triangulation to enhance credibility as our research team has diverse expertise in pediatric palliative care, palliative care, health technology and ethics. | This approach was followed almost to the point with the exception that BH controlled and oversaw the development of themes |
